# No association of natural killer cell number and function in peripheral blood with overweight/obesity and metabolic syndrome in a cohort of young women

**DOI:** 10.14814/phy2.15148

**Published:** 2022-02-18

**Authors:** Julia Keilen, Christina Gar, Marietta Rottenkolber, Louise U. Fueessl, Anna T. Joseph, Rika Draenert, Jochen Seissler, Andreas Lechner

**Affiliations:** ^1^ Diabetes Research Group Department of Medicine IV University Hospital LMU Munich Munich Germany; ^2^ Clinical Cooperation Group Diabetes Ludwig‐Maximilians‐Universität München and Helmholtz Zentrum München Munich Germany; ^3^ German Center for Diabetes Research (DZD) München‐Neuherberg Germany; ^4^ Stabsstelle Antibiotic Stewardship LMU Klinikum Munich Munich Germany

## Abstract

**Aim:**

To reexamine the associations of NK cell number and function in the peripheral blood with overweight/obesity and the metabolic syndrome in a large, well‐phenotyped human cohort.

**Methods:**

Cross‐sectional analysis of 273 women in the PPSDiab Study; measurement of absolute and relative number of NK cells in peripheral blood, and of functional parameters CD69 positivity and cytotoxicity against K562 cells; group comparison of NK cell characteristics between lean, overweight, and obese participants, as well as metabolic syndrome scores of 0, 1, 2, and ≥3; Spearman correlation analyses to clinical parameters related to the metabolic syndrome.

**Results:**

We found no differences in NK cell number and function between lean, overweight, and obese women (relative NK cell number (median (Q1–Q3), [%]) 5.1(2.6–9.4) vs. 4.8 (2.9–8.4) vs. 3.8 (1.7–7.8), *p* = 0.187; absolute NK cell number [10^6^/L]: 86.9 (44.6–188.8) vs. 92.6 (52.5–154.6) vs. 85.9 (44–153.8), *p *= 0.632; CD69+ [%]: 27.2 (12.9–44.3) vs. 37.6 (13.2–52.8) vs. 33.6 (16.3–45), *p *= 0.136; cytotoxicity [%]: 11.0 (7.1–14.5) vs. 8.5 (6.4–13.2) vs. 11.3 (8.7–14.2), *p *= 0.094), as well as between different metabolic syndrome scores. Nonesterified fatty acids correlated with absolute and relative NK cell number and cytotoxicity (*ρ* [*p*‐value]: 0.142 [0.021], 0.119 [0.049], and 0.131 [0.035], respectively). Relative NK cell number further correlated with high‐density lipoprotein cholesterol (0.144 [0.018]) and cytotoxicity with 2 h glucose in oral glucose tolerance testing (0.132 [0.034]). CD69 positivity correlated with body fat (0.141 [0.021]), triglycerides (0.129 [0.033]), and plasma leptin (0.155 [0.010]). After correction for multiple testing, none of the associations remained significant.

**Conclusion:**

In the present study, we observed no associations of NK cell number and function in the peripheral blood with overweight/obesity and the metabolic syndrome. Extreme phenotypes of obesity and the metabolic syndrome might have caused differing results in previous studies. Further analyses with a focus on compartments other than peripheral blood may help to clarify the relation between NK cells and metabolic diseases.

## Graphical abstract

Contrary to previous, population‐based studies, NK cell number and function in the peripheral blood are not altered with overweight/obesity or the metabolic syndrome in a cohort of premenopausal women. Obesity‐associated immune dysfunction may therefore be less pronounced in this segment of the population.

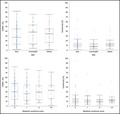

## 1 INTRODUCTION

Obesity, the metabolic syndrome, and the immune system are linked in several ways. For example, immune cell infiltration into adipose tissue intensifies the metabolic syndrome (Sell et al., 2012). A specific part of the immune system implicated in this cross talk is natural killer (NK) cells, a component of innate immunity.

In obese mice, the secretion of IFN‐γ and TNF‐α by NK cells promoted macrophage infiltration into the adipose tissue (Bonamichi & Lee, 2017; Wensveen et al., 2015). The so‐called M2‐to‐M1 transition that turns anti‐inflammatory into pro‐inflammatory macrophages finally promotes insulin resistance of the visceral adipose tissue linking obesity to type 2 diabetes in the context of the metabolic syndrome (Wensveen et al., 2015). Consistent with this finding, the ablation of NK cells leads to an increased insulin sensitivity (O'Rourke et al., 2014). Additionally, diet‐induced obese mice exhibit reduced NK cell cytotoxicity with an increased mortality rate of the mice when compared to their lean counterparts (Smith et al., 2007).

In humans, several studies reported a reduced number (Lynch et al., 2009; O'Shea et al., 2010; Rodriguez et al., 2018; Tobin et al., 2017), impaired function (Jung et al., 2018; Kim et al., 2017; O'Shea et al., 2010; Tobin et al., 2017), or higher activation of peripheral NK cells (Lynch et al., 2009; O'Rourke et al., 2013; Tobin et al., 2017; Viel et al., 2017) with obesity and the metabolic syndrome and these alterations were found to normalize with weight loss (Jahn et al., 2015; Moulin et al., 2011). Similarly, adipose tissue NK cells of obese subjects exhibit an increased number of activation markers (O'Rourke et al., 2013). Consequently, NK cells might favor a chronic inflammatory state and thus be crucial player in the development of the metabolic syndrome in obesity. However, the cohorts under investigation were relatively small and the grades of obesity examined were often extreme. Additionally, the non‐standardized methodology for measuring NK cell function impedes the comparison of different study results.

Besides the metabolic syndrome, deficits in NK cell number and function have also been implicated in the development and progression of cancer (Imai et al., 2000; Michelet et al., 2018). These cells could thus provide an explanation for the increased incidence of certain cancers with obesity and the metabolic syndrome if, in fact, deficits of cell number and function were consistent under these conditions.

Here, we reexamined the associations of NK cell number and function in the peripheral blood with overweight/obesity and characteristics of the metabolic syndrome in a large, well‐phenotyped human cohort. We studied young women after a pregnancy with or without gestational diabetes mellitus, because this cohort incorporates a wide range of adiposity and metabolic states but little confounding factors (Gar et al., 2017). We measured the relative and absolute NK cell number, activation via CD69 positivity, and cytotoxicity in a killing assay.

## 2 MATERIALS AND METHODS

### 2.1 Study cohort

The study participants were part of the prospective, monocenter observational study “Prediction, Prevention and Subclassification of gestational and type 2 Diabetes” (PPSDiab) (Rottenkolber et al., 2015). Women were recruited from the diabetes center and the obstetrics department of the University Hospital in Munich (LMU Klinikum), Germany. They were included between November 2011 and May 2016. The cohort consists of women, who had been diagnosed with gestational diabetes (GDM) in a recent pregnancy and a group of women with a normoglycemic pregnancy in a ratio of 2:1, giving live birth to singletons (*n* = 295) or twins (*n* = 9). The study visit was conducted 3–16 months after delivery.

The GDM diagnosis was based on a 75 g of oral glucose tolerance test (OGTT) after the 23rd gestation week. Cut‐off values were ≥92 mg/dl after fasting, ≥180 mg/dl after 1 h, and ≥153 mg/dl after 2 h, according to the recommendations of the International Association of the Diabetes and Pregnancy Study Groups (IADPSG) (Metzger et al., 2010). Normoglycemia during pregnancy was confirmed by an OGTT after the 23rd week of gestation either with 75 g of glucose (*n* = 294; cut‐off values for GDM according to the IADPSG) or with a 50 g of glucose screening test (*n* = 10; plasma glucose after 1 h <135 mg/dl to confirm normoglycemia).

Exclusion criteria of the PPSDiab study were substance or alcohol abuse, a diagnosis of diabetes prior to the pregnancy, and chronic diseases requiring long‐term medication. As an exception we included women with medication for hypothyroidism (*n* = 52), mild hypertension (*n* = 4), gastro‐esophageal reflux (*n* = 2), and one participant receiving rivaroxaban prophylaxis after pulmonary embolism.

Written informed consent was obtained from all study participants, and the protocol was approved by the ethical review committee of the Ludwig‐Maximilians‐Universität (study ID 300‐11).

### 2.2 Groups

We excluded four women from the baseline visit of the PPSDiab study due to acute upper respiratory infection at the study visit (*n* = 1), overt hyperthyroidism (*n* = 2), and diagnosis of T1D at the baseline visit (*n* = 1). From the remaining 300 participants, a valid NK cell isolation was available for 280 participants. Furthermore, seven women were excluded from the analysis due to a missing value for the waist circumference (*n* = 6) or BMI (*n* = 1). Consequently, the final study sample consisted of 273 study participants (Figure S1 [https://figshare.com/s/6e068152ed48514cb38c]).

Overweight was defined as a BMI of at least 25 kg/m^2^ and obesity as a BMI of at least 30 kg/m^2^. The metabolic syndrome score was calculated as the sum of criteria met from the five NCEP ATPIII criteria for the metabolic syndrome (Alberti et al., 2009), that is, (Goossens et al., 2020) an increased waist circumference with >102 cm for men or >88 cm for women, (Sell et al., 2012) blood pressure ≥ 130/85 mm Hg, (Wensveen et al., 2015) fasting triglycerides (TG) ≥ 150 mg/dl, (Bonamichi & Lee, 2017) fasting high‐density lipoprotein (HDL) cholesterol < 40 mg/dl for men or <50 mg/dl for women, and (O'Rourke et al., 2014) an increased fasting glucose ≥ 100 mg/dl.

### 2.3 Measurements

The samples for this analysis were drawn at the baseline visit of the PPSDiab study, which was conducted 3–16 months after the index pregnancy. For the blood drawing, the women were fasted for at least 8 h and had no signs of an acute illness. Blood lipids (triglycerides, low‐density lipoprotein (LDL) cholesterol, and high‐density lipoprotein (HDL) cholesterol; enzymatic caloric test; Roche Diagnostics), high‐sensitivity C‐reactive protein (hsCRP; wide‐range C‐reactive protein; Siemens Health Care Diagnostics), plasma leptin (ELISA "Dual Range," Merck Millipore), serum nonesterified fatty acids (NEFA; enzymatic calorimetric method, NEFA Kit, Wako Chemicals), and the differential blood count were determined in the fasted state.

Additionally, all women underwent a standardized 75 g of 5‐point OGTT (0, 30, 60, 90, and 120 min) with measurements of plasma glucose (Glucose HK Gen.3; Roche Diagnostics) and serum insulin (chemiluminescent immunoassay; DiaSorin LIASON Systems) as described in detail elsewhere (Gar et al., 2017). From the OGTT, the insulin sensitivity index (ISI) was calculated according to Matsuda and DeFronzo (Matsuda & DeFronzo, 1999).

Blood pressure was measured twice within an at least 30‐min interval in a resting sitting position and the mean out of the two measurements was used for the present analyses. Height and waist circumference were determined to the nearest 1 cm. Weight and total body fat were measured by a bioelectrical impedance analysis scale (Tanita BC‐418; Tanita Corporation) (Bosy‐Westphal et al., 2008, 2013). Further information about the visit conduct can be found elsewhere (Rottenkolber et al., 2015).

### 2.4 Preparation of peripheral blood mononuclear cells (PBMC)

PBMC were isolated from 7.5 ml of fresh EDTA blood (S‐Monovette 7.5 ml of K3 EDTA; Sarstedt) within 30 h by density gradient centrifugation using the Hypaque‐Ficoll method (Ferrante & Thong, 1980). Finally, the PBMC were mixed with 2 ml of dimethyl sulfoxide (DMSO; Carl Roth GmbH +Co. KG) containing 10% of fetal calf serum (FCS, FBS Superior; Biochrom, Cambourne) and stored in liquid nitrogen until assayed.

### 2.5 Flow cytometric analysis

PBMC were thawed and incubated 1 day before the phenotypic and functional analyses (Draenert et al., 2004). Thawed PBMC were counted using trypan blue (Crowley et al., 2016) and resuspended in RPMI to a concentration of 10^6^ cells per ml. All following reagents had a temperature of 4°C and steps containing antibodies were conducted in the dark.

The PBMC (2.5 10^5^) were added to 2 ml of phosphate‐buffered saline (PBS, Dulbecco's Phosphate‐Buffered Saline; Sigma‐Aldrich) with 1% FCS to prevent nonspecific background staining (Lalor and Revell, 1989). A centrifugation of 8 min at 491 g ensued. For phenotypic analysis, the cell pellet was mixed with 4 μl of BD Simultest^TM^ CD3/CD16+CD56. This reagent for determining NK cells contains FITC‐labeled CD3 (SK7), PE‐labeled CD16 (B73.1), and PE‐labeled CD56 (MY31). We further added 16 μl of PerCP‐labeled CD45 (2D1), which is a marker generally found on leukocytes (O'Shea et al., 2010; Rheinlander et al., 2018) and 20 μl of APC‐labeled CD69 (FN50) as a marker for activation (Lynch et al., 2009; Tobin et al., 2017; Viel et al., 2017). Isotype antibodies served as controls. All antibodies were obtained from Becton Dickinson (BD) Biosciences. Required amounts of antibodies were determined in a former titration series.

After incubation for 30 min, cells were washed with PBS and fixed with Fix/Perm Solution A (Caltag Medsystems Ltd).

Flow cytometric analyses were conducted using a FACSCalibur (BD Biosciences) and the FlowJo software (version 10.5.3; BD Biosciences). Gating was performed by two independent raters (JK and CG). If the mean value was out of the limits of agreement, the gating was evaluated by a third person (AL).

To examine the number of NK cells, we first gated the lymphocytes using forward side scatter (FSC) and sideward side scatter (SSC) (Figure S2A [https://figshare.com/s/6e068152ed48514cb38c]). We further validated the quality of the lymphocyte gate by acquiring the CD45^+^ lymphocytes (O'Shea et al., 2010) (Figure S2B [https://figshare.com/s/6e068152ed48514cb38c]). NK cells were defined as CD3^−^ and CD56^+^ and/or CD16^+^ (Fraker & Bayer, 2016; Lanier et al., 1986; Shi et al., 2011) and were expressed as a percentage of CD45^+^ lymphocytes (Figure S2C [https://figshare.com/s/6e068152ed48514cb38c]). Besides, we analyzed the proportion of CD69^+^ NK cells among all NK cells (Figure S2D [https://figshare.com/s/6e068152ed48514cb38c]).

To examine the absolute number of NK cells ${\text{NK}}_{\text{abs}}$, we multiplied the percentage of relative NK cells ${\text{NK}}_{\text{rel}}$ with the amount of lymphocytes per μl in the differential blood count ${\text{lymph}}_{\text{abs}}$ $\left({\text{NK}}_{\text{abs}}={\text{NK}}_{\text{rel}}\cdot {\text{lymph}}_{\text{abs}}\right)$.

### 2.6 NK cell function assay

Cytotoxicity of NK cells was examined using the NKTEST™ reagent kit (BD Biosciences) according to the manufacturer's instruction. It contains cryopreserved, green fluorescent pre‐stained K562 cells (human erythroleukemia cell line commonly used as a target for NK cells) (Dovio et al., 2004; Lozzio, 1975; Mhatre et al., 2014).

Afterward, PBMC (effector cells; E) were mixed with K562 cells (target cells; T) at different E:T ratios ranging from 2.5:1 to 200:1 and incubated them for 2 h in a CO_2_ incubator at 37°C. As a positive control for lysis, we added 20 μl of saponin at 0.3 mg/ml to K562 cells as described by Piriou et al. (Piriou et al., 2000). To determine spontaneous death of PBMC and K562 cells and as negative controls we also incubated them separately.

After incubation, we added 30 μl of red fluorescent DNA staining solution from the NKTEST kit to each sample and measured them within 30 min. We conducted the gating for the function assay in the histogram view as described by the manufacturer. Based on the green and red fluorescent dye, we were able to distinguish between dead tumor cells K562 (red + green), living K562 (green), dead PBMC (red), and living PBMC (none).

First, we gated the green dyed K562 cells in the negative control and transferred the gate to all samples. We ensured that less than 1.5% of PBMC were included in that gate. Then we set a gate on dead K562 cells and again transferred it to all samples. Thereby, the percentage of dead K562 among all K562 cells was determined for each E:T ratio. Accordingly, we identified the spontaneous death of PBMC and K562 cells in the negative controls as well as the proportion of dead K562 cells in the positive control.

To adjust for possible pipetting inaccuracies, we calculated the factual E:T ratios for every sample as ${\text{Ratio}}_{E:T}=\frac{K562_{\text{con}}}{K562_{\text{sam}}}$ (with $K562_{\text{sam}}$ as the proportion of green dyed K562 from all cells in the samples and $K562_{\text{con}}$ as the proportion of green dyed K562 from all cells in the negative control).

Together with ${\text{NK}}_{\text{rel}}$, we determined the ratio of NK cells to T in each sample as ${\text{Ratio}}_{E_{\text{NK}}:T}={\text{NK}}_{\text{rel}}\cdot {\text{Ratio}}_{\text{E:T}}$.

The amount of killed cells was computed by subtracting the spontaneous K562 cell death from the proportion of killed tumor cells in the different ratio samples.

To evaluate the cytotoxicity of the NK cells (measured as killing capacity toward K562 cells), we plotted the ${\text{Ratio}}_{E_{\text{NK}}:T}$ against the proportion of killed cells and obtained a graph for every study participant. For every graph, we calculated the slope of the association between the proportion of killed cells and the ${\text{Ratio}}_{E_{\text{NK}}:T}$ as representations for the function of the NK cells (Figure S3 [https://figshare.com/s/6e068152ed48514cb38c]).

### 2.7 Statistical analysis

All metric and normally distributed variables are reported as mean ± standard deviation; non‐normally distributed variables are presented as median (first quartile–third quartile). Group comparisons were conducted using the Kruskal–Wallis test. Spearman correlation coefficients (ρ) were calculated for correlation analyses. *p*‐values <0.05 were considered statistically significant and correction for multiple testing was achieved by the Bonferroni method. All statistical calculations were performed using SAS statistical software package, version 9.4 (SAS Institute Inc., Cary, NC, USA). Supplemental Figures were created using Microsoft^®^ PowerPoint^®^ (version 2101) based on outputs from FlowJo software (Figure S2; version 10.5.3; BD Biosciences) and R (Figure S3; version 3.1.3, R Development Core Team).

## 3 RESULTS

A total of 273 participants with a characterization of NK cells, measurement of BMI, and complete data on the metabolic syndrome score were available for the present analyses (Figure S1 [https://figshare.com/s/6e068152ed48514cb38c]). In 7 women, no data on lymphocyte count in whole blood were available, which restricted the calculation of the absolute number of NK cells to 266 women. Additionally, in 15 samples, the total number of isolated PBMC was insufficient for the determination of cytotoxicity. Hence, cytotoxicity was only available for 258 participants.

Baseline characteristics of the study cohort are presented in Table 1. The mean ± SD and range (min–max) of BMI in the lean, overweight, and obese group were 21.8 ± 1.7 kg/m^2^, 26.6 ± 1.3 kg/m^2^, and 36.1 ± 5.0 kg/m^2^, and 17.47–24.94 kg/m^2^, 25.04–29.97 kg/m^2^, and 30.0–49.87 kg/m^2^, respectively.

**TABLE 1:** Baseline characteristics of the PPSDiab study cohort (*n* = 273)

	Distribution	Range
Age (year)	35.5 ± 4.3	20–49
BMI (kg/m^2^)	25.4 ± 5.9	17.5–49.9
Waist circumference (cm)	81.3 ± 11.4	63–120
Body fat (%)	32.2 ± 8.0	12.5–53.5
Systolic blood pressure (mm Hg)	117.6 ± 11.7	94–156
Diastolic blood pressure (mm Hg)	73.8 ± 9.2	50–107
Triglycerides (mg/dl)	67 (53–92)	27–313
HDL cholesterol (mg/dl)	62 (54–72)	32–127
LDL cholesterol (mg/dl)	104 (86–121)	32–214
NEFA (µmol/L)	591 (467–742)	51–1415
Leptin (ng/ml)	10.6 (5.6–16.2)	0.7–78.3
hsCRP	0.05 (0.01–0.17)	0–2.1
Fasting plasma glucose (mg/dl)	92.8 ± 9.2	65–136
Two hour plasma glucose (mg/dl)	113.2 ± 31.8	53–241
ISI	5.2 (3.4–7.5)	0.8–18.2
History of gestational diabetes (*n* [%])	176 (64.5)	—
Pathological glucose tolerance (*n* [%])	75 (27.5)	—
Leukocytes (10^9^/L)	5.6 (4.8–6.6)	2.6–11.6
Lymphocytes (%)	34 (28–38)	13–57
NK cells (% lymphocytes)	4.8 (2.5–9.1)	0.2–41.3
NK cells (10^6^/L)	86.8 (45.4–165.4)	45–8997.4
CD69^+^ (% NK cells)	29.2 (13.1–45.4)	1.5–82.5
Cytotoxicity (%)	10.6 (7.0–14.4)	1.8–94.4

Note

Distribution is given as mean ± SD, median (Q1–Q3) or frequency (%) (middle panel); range is given as minimum–maximum value (right panel). Body fat: missing *n* = 2; ISI: missing *n* = 1; leukocytes: missing *n* = 1; lymphocytes: missing *n* = 7; NK cell number: missing *n* = 7; cytotoxicity: missing *n* = 15.

Abbreviations: BMI, body mass index; CD69^+^, positive for cluster of differentiation 69; HDL, high‐density lipoprotein; hsCRP, high‐sensitivity C‐reactive protein; ISI, insulin sensitivity index; LDL, low‐density lipoprotein; NEFA, nonesterified fatty acids; NK cells, natural killer cells.

**FIGURE 1:**
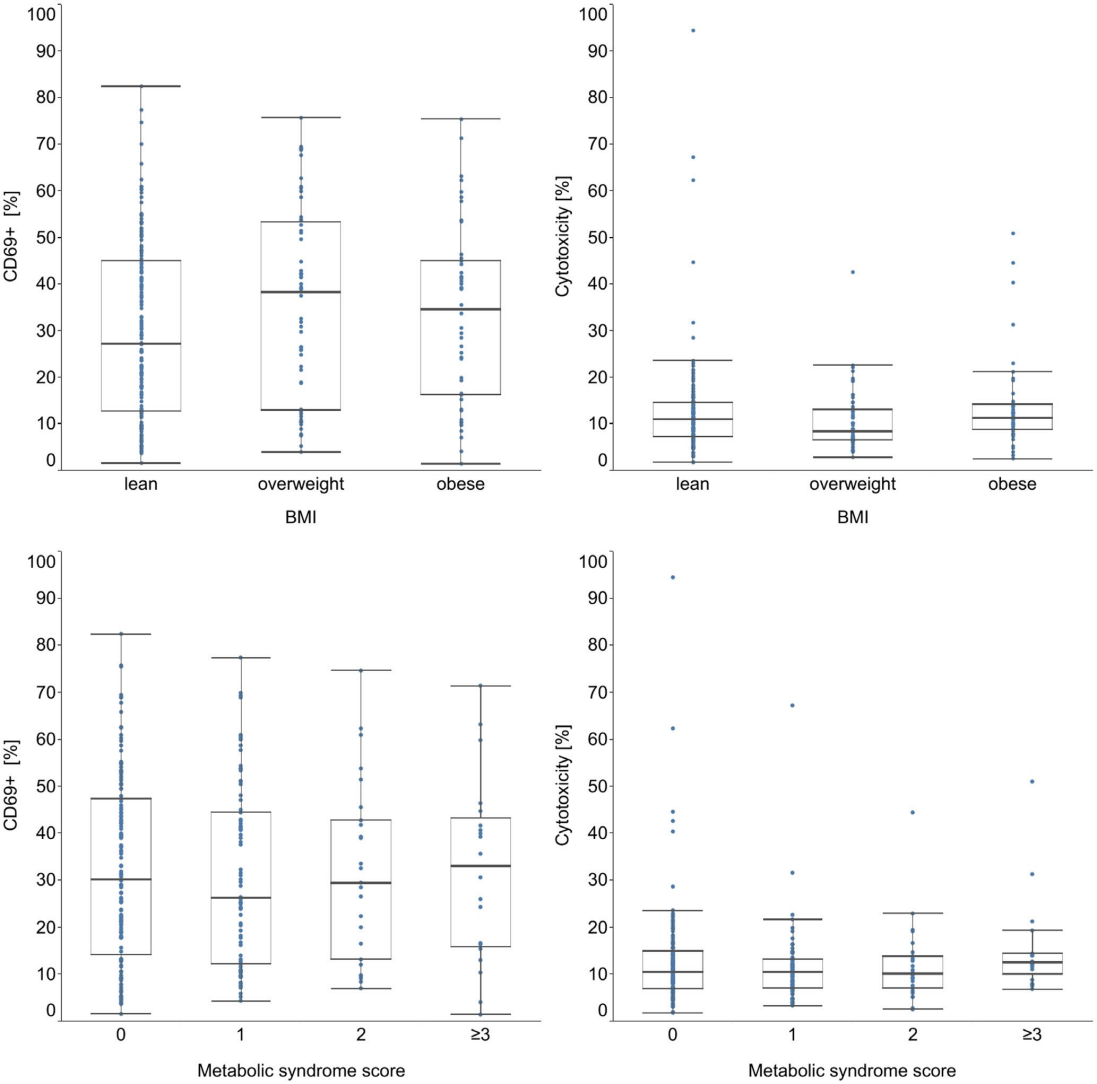
Group comparison of CD69 positivity (left panels) and cytotoxicity (right panels) between BMI categories (upper panels) and increasing metabolic syndrome scores (lower panel). Groups displayed no significant differences in proportion of CD69+ NK cells and cytotoxicity (Kruskal–Wallis test *p* > 0.05). Due to small group sizes for a metabolic syndrome score of 4 and 5, women with a score of ≥3 were analyzed as one group

We first examined the association of NK cell characteristics with overweight and obesity. In this examination, we found no significant difference in NK cell characteristics between lean, overweight, and obese women (Figure 1, Table 2).

**TABLE 2:** Group comparison of NK cell characteristics between lean, overweight, and obese women

BMI group	Lean	Overweight	Obese	*p*‐value
*n*	165	61	47	
NK cells (% lymphocytes)	5.1 (2.6–9.4)	4.8 (2.9–8.4)	3.8 (1.7–7.8)	0.187
NK cells (10^6^/L)	86.9 (44.6–188.8)	92.6 (52.5–154.6)	85.9 (44–153.8)	0.632
CD69+ (% NK cells)	27.2 (12.9–44.3)	37.6 (13.2–52.8)	33.6 (16.3–45)	0.136
Cytotoxicity (%)	11.0 (7.1–14.5)	8.5 (6.4–13.2)	11.3 (8.7–14.2)	0.094

Note

Group comparison with the Kruskal–Wallis test. Values are given as median (Q1–Q3). NK cell number: missing *n* = 7; cytotoxicity: missing *n *= 15.

Our second research question was whether NK cell characteristics were associated with the metabolic syndrome. The number of women with four (*n* = 4) or five (*n* = 1) points for the metabolic syndrome score according to the NCEP ATPIII criteria was too small to constitute an own group. Therefore, we compared NK cell characteristics between women with 0, 1, 2, and ≥3 points for the metabolic syndrome. None of the NK cell characteristics showed significant differences between these groups (Figure 1, Table 3).

**TABLE 3:** Group comparison of NK cell characteristics between women at varying degrees of the metabolic syndrome

Metabolic syndrome score	0	1	2	≥3	*p*‐value
*n*	146	82	25	20	
NK cells (% lymphocytes)	4.8 (2.4–8.7)	5.4 (3.1–10.0)	4.8 (2.3–7.7)	3.5 (1.4–8.4)	0.277
NK cells (10^6^/L)	84.4 (44.1–140.9)	112.1 (46.1–196.2)	83.5 (46.6–154.3)	86.4 (23.7–195.8)	0.971
CD69+ (% NK cells)	30.3 (14.8–47.3)	26.4 (12.2–44.4)	29.5 (13.1–42.7)	33.1 (15.8–43.2)	0.495
Cytotoxicity (%)	10.4 (6.9–14.7)	10.4 (7.0–13.2)	10.1 (7.0–13.7)	12.5 (8.9–14.5)	0.267

Note

Group comparison with the Kruskal–Wallis test. Values are given as median (Q1–Q3). NK cell number: missing *n* = 7; cytotoxicity: missing *n* = 15.

Finally, we calculated exploratory Spearman correlation coefficients of the different NK cell characteristics with components of the metabolic syndrome and associated laboratory and anthropometric characteristics (Table 4). Here, the absolute NK cell number correlated with HDL cholesterol and NEFA and the relative NK cell number correlated with HDL only. The amount of activated NK cells (CD69^+^) correlated with body fat, serum triglycerides, and plasma leptin. Cytotoxicity displayed correlations with NEFA and the 2 h glucose value in the OGTT. All correlations were positive and only HDL cholesterol with relative NK cell number remained significant after correction for multiple testing.

**TABLE 4:** Exploratory correlation analysis of NK cell characteristics with selected characteristics of the metabolic syndrome (*n* = 273)

	NK cells (% lymphocytes)		NK cells (10^6^)		CD69+ (% NK cells)		Cytotoxicity (%)	
	*ρ*	*p*‐value	*ρ*	*p*‐value	*ρ*	*p*‐value	*ρ*	*p*‐value
Age	−0.002	0.976	−0.040	0.519	0.019	0.754	−0.005	0.936
BMI	−0.085	0.163	−0.016	0.801	0.101	0.095	−0.039	0.538
Waist circumference	−0.063	0.296	0.002	0.977	0.041	0.503	0.014	0.817
Body fat	−0.104	0.087	−0.042	0.498	**0.142**	**0.019**	−0.025	0.695
Systolic blood pressure	−0.046	0.450	−0.031	0.617	0.054	0.373	0.028	0.660
Diastolic blood pressure	0.022	0.716	0.025	0.688	0.051	0.403	0.034	0.589
Triglycerides	−0.074	0.225	−0.014	0.823	**0.128**	**0.034**	0.043	0.495
HDL cholesterol	**0.211**	**<0.001**	**0.138**	**0.024**	−0.032	0.594	−0.007	0.906
LDL cholesterol	−0.010	0.872	0.027	0.658	0.077	0.206	0.025	0.684
NEFA	0.105	0.083	**0.124**	**0.043**	−0.041	0.502	**0.187**	**0.003**
Leptin	−0.059	0.332	0.004	0.949	**0.136**	**0.025**	−0.022	0.727
hsCRP	−0.066	0.279	−0.011	0.856	0.104	0.088	0.029	0.641
Fasting glucose	0.039	0.518	0.047	0.446	−0.005	0.932	0.107	0.088
Two hour glucose	−0.051	0.399	0.006	0.925	−0.072	0.235	**0.182**	**0.003**
ISI	0.062	0.305	−0.003	0.958	−0.044	0.465	−0.062	0.322

Note

Spearman correlation coefficients. Correlations with exploratory significance (*p* < 0.05) are marked in bold. NK cell number: missing *n* = 7; cytotoxicity: missing *n* = 15; body fat: missing *n* = 2; ISI: missing *n* = 1.

Abbreviations: BMI: body mass index; HDL: high‐density lipoprotein; hsCRP: high‐sensitivity C‐reactive protein; ISI: insulin sensitivity index; NEFA: nonesterified fatty acids; NK cells: natural killer cells.

## 4 DISCUSSION

In the present study, we observed no associations of NK cell number and function in the peripheral blood with overweight/obesity and the metabolic syndrome in a cohort of young women. However, we detected several correlations of NK cell parameters with metabolic characteristics, but these were weak and inconclusive with respect to physiologic context.

Based on previous studies, we had anticipated a reduced NK cell number, higher activation, and lower cytotoxicity with overweight/obesity and the metabolic syndrome. However, this was not the case in our analysis. If anything, we observed weak trends toward a lower relative NK cell number and higher activation with increasing body adiposity, but the relevance of these observations remains questionable. Because NK cell number and activation can be measured robustly through flow cytometry, differences in these parameters between previous studies and ours are probably not due to the measurement technique (Ryder et al., 2014). PBMC preparation and storage should also not affect these parameters significantly, which leaves the structure of the different cohorts as the most likely reason for observing divergent results. Most cohorts that studied NK cells in the context of BMI relied on morbid obesity, with a BMI ranging from about 40 to over 50 kg/m^2^ (Laue et al., 2015; O'Rourke et al., 2013; O'Shea et al., 2010; Viel et al., 2017). Additionally, previous studies often had small group sizes (Bahr et al., 2018; Laue et al., 2015; Moulin et al., 2011; O'Rourke et al., 2013; Viel et al., 2017) or examined individuals who underwent a drastic intervention, for example, bariatric surgery (Jahn et al., 2015; Moulin et al., 2011; O'Rourke et al., 2013). In the comparison of the different studies, the respective ways to calculate and present NK cell numbers must also be taken into account. One study by Rodriguez et al., which is most comparable to ours, was conducted in a cohort with a rather normal range of BMI, a sufficient number of subjects, and deep phenotyping (Rodriguez et al.,). This study also found a reduced NK cell number with higher body fat but only reported the proportion of NK cells from all lymphocytes, not the absolute number. However, an increase in total lymphocytes with higher BMI has already been shown by others (Ryder et al., 2014) and this tendency can even be observed in our cohort, despite moderate BMI levels. Therefore, a relative decrease in NK cells with higher BMI does not necessarily indicate a change in the absolute NK cell number in the peripheral blood.

For cytotoxicity, the situation is more complex. Flow cytometry‐based killing assays are now the standard measurement technique for this NK cell characteristic, though these assays are conducted and read differentially between studies. For example, some previous studies relied on a single E:T ratio (Berrou et al., 2013; Imai et al., 2000; Tobin et al., 2017; Viel et al., 2017) and others did not quantify the true E:T ratio but rather relied on the ratio achieved theoretically through the pipetted volumes (Dovio et al., 2004; Kim et al., 2017; O'Shea et al., 2010; Smith et al., 2007). Instead, we relied on several E:T ratios for each participant and quantified the final ratio of each sample. We then calculated an index of cytotoxicity per NK cell for each study participant. Thus, our approach may have provided a less biased view on NK cell cytotoxicity compared to previous studies.

Among the observed correlations of NK cell parameters with metabolic characteristics, those of NK cell activation with body fat, triglycerides, and leptin seem most consistent with previous work. For example, Viel et al. also found more CD69+ NK cells in obese compared to nonobese individuals and suggested chronic overstimulation of NK cells in the context of metabolic inflammation as the underlying mechanism (Viel et al., 2017). However, in our analysis, body fat and other signs of the metabolic syndrome did not display correlations of comparable magnitude with NK cell activation. Further correlations we observed were also weak and inconclusive with respect to their physiologic context. For example, relative NK cell number correlated positively with HDL cholesterol, a parameter indicating metabolic health, as well as NEFA, which have been linked to obesity, insulin resistance, and the metabolic syndrome (Guilherme et al., 2008; Kahn et al., 2006; McGarry, 2002). Only the correlation between HDL cholesterol relative and NK cell number remained significant after correction for multiple testing but is difficult to interpret in the context of our study and the literature. The remaining correlations did not meet stringent criteria of significance. Therefore, they may well be a consequence of multiple testing and not true observations.

A main strength of our study is its large, deeply phenotyped cohort of young individuals of the same sex with little comorbidities. Furthermore, we performed our NK cell analyses to a high‐quality standard. First, we included a CD45 antibody to validate the lymphocyte gate (O'Shea et al., 2010). Besides, gating was performed by 2–3 independent assessors. Second, we measured the number of NK cells truly included in the individual killing assays. Thereby, we were able to exclude errors secondary to unbalanced cell distribution, which easily occur otherwise. Finally, instead of relying on a single E:T ratio, we determined the killing ability of NK cells in a wide range of ratios (2.5:1–200:1).

A significant limitation of our study is the focus on one function of NK cells only, namely the ability to kill cells. This impedes the comparison to other studies that examined other functions of NK cells, for example, IFN‐γ secretion, CD107a expression, or lactate dehydrogenase (LDH) activity (Bahr et al., 2018; Dovio et al., 2004; Kim et al., 2019). Also, we did not investigate further subtypes of NK cells (i.e., CD16/56 dim/bright). In this context, the cytolytic profile and the proportion of CD56 dim NK cells were found to change during pregnancy and the early postpartum period (Kraus et al., 2012). Hence, we cannot fully exclude that in our cohort NK cell function was affected by the recent pregnancy. Furthermore, the small number of participants with pertinent metabolic syndrome entails a low sensitivity regarding the analysis of NK cell alterations with the metabolic syndrome. Lastly, our specific, homogenous cohort may have led to the observed negative result and a population‐based sample may have been more revealing.

## 5 CONCLUSION

Our study does not confirm a role of NK cell number and function in the peripheral blood as cause or consequence of overweight/obesity and the metabolic syndrome. The cohort under investigation and our choice of measurements may have caused this negative result. Nevertheless, alteration of NK cell characteristics with metabolic diseases does not seem to be a universal feature. Further analyses with a focus on compartments other than peripheral blood may help to clarify the relation between NK cells and metabolic diseases.

## CONFLICT OF INTEREST

The authors have nothing to disclose.

## AUTHOR CONTRIBUTIONS

Conceptualization, J.K., C.G., R.D., and A.L.; Formal Analysis, J.K., C.G., and M.R.; Investigation J.K., C.G., L.F., and A.J.; Data Curation, C.G. and M.R..; Writing – Original Draft, J.K. and C.G.; Writing – Review & Editing, all; Visualization, C.G.; Supervision, R.D., J.S., and A.L.; Project Administration A.L.; Funding Acquisition A.L. and J.S.; A.L. is the guarantor of this work. This work was not published previously.

## Supporting information



Supplementary Material
