# Supplementary material for: No association of natural killer cell number and function in peripheral blood with overweight/obesity and metabolic syndrome in a cohort of young women
Source: Physiol Rep. 2022 Feb 18;10(4):e15148. doi: 10.14814/phy2.15148 (PMC8855889; doi:10.14814/phy2.15148)

**Supplementary figures**

**No association of natural killer cell number and function in peripheral blood with overweight/obesity and metabolic syndrome in a cohort of young women**

Julia Keilen* ^123^, Christina Gar* ^123^, Marietta Rottenkolber ^123^, Louise U Fueessl ^123^, Tina Joseph ^123^, Rika Draenert ^4^, Jochen Seissler ^123^, Andreas Lechner ^123^

*shared first authors

^1^ Diabetes Research Group, Department of Medicine IV, University Hospital, LMU Munich, Germany;

^2^ Clinical Cooperation Group Diabetes, Ludwig-Maximilians-Universität München and Helmholtz Zentrum München, Munich, Germany;

^3^ German Center for Diabetes Research (DZD), München-Neuherberg, Germany;

^4^ Stabsstelle Antibiotic Stewardship, LMU Klinikum Munich, Germany.

**Fig. S1**: **Flow chart of the study cohort.** NK: natural killer; T1D: type 1 diabetes mellitus.

**Fig. S2**: **Gating strategy for the determination of lymphocytes (A and B), the NK cell subset (C), and CD69+ NK cells (D).** NK: natural killer.


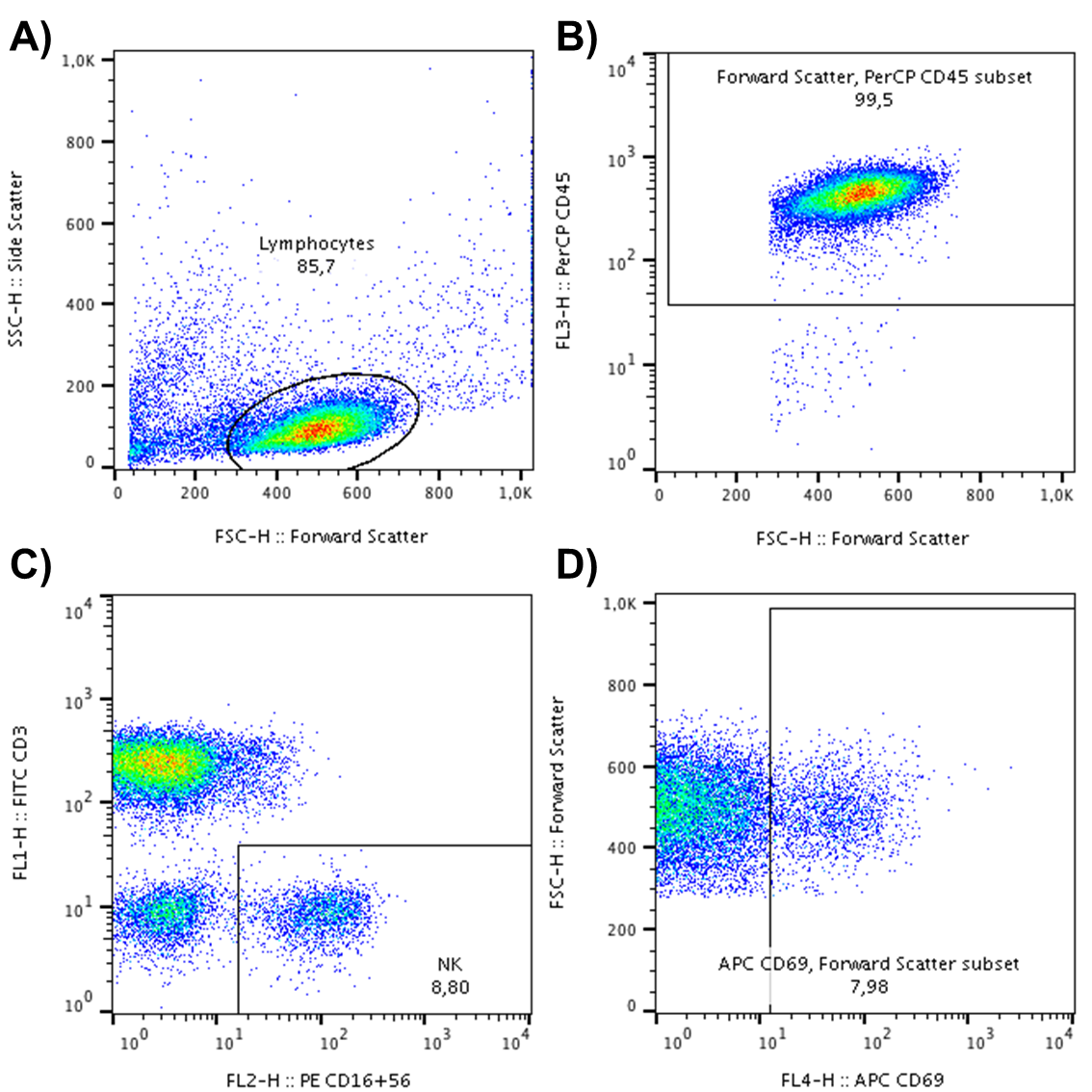


**Fig. S3**: **Determination of the killing-assay slope (exemplary).** Black dots: proportion of killed K562 cells at the measured effector-to-target (E:T)-ratio; blue line: determined slope; grey shaded are: 95% CI of the slope.


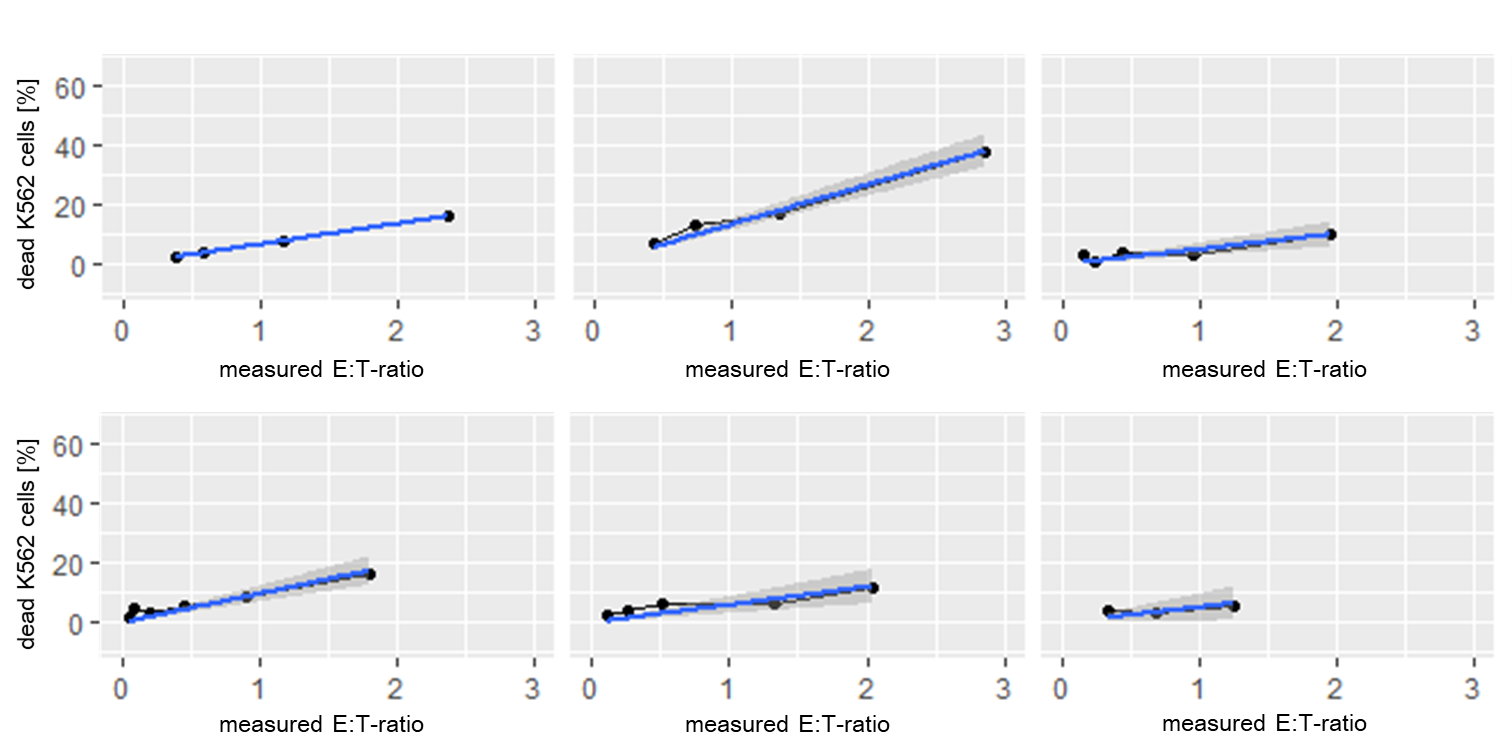

Supplement: Supplementary file 1 — Supplementary Material [file PHY2-10-e15148-s001.docx]
